# Boron Trifluoride Anionic Side Groups in Polyphosphazene Based Polymer Electrolyte with Enhanced Interfacial Stability in Lithium Batteries

**DOI:** 10.3390/polym10121350

**Published:** 2018-12-05

**Authors:** Sebastian Schmohl, Xuan He, Hans-Dieter Wiemhöfer

**Affiliations:** Institute of Inorganic and Analytical Chemistry, University of Münster, Corrensstraße 28/30, 48149 Münster, Germany; sebastian.schmohl@uni-muenster.de (S.S.); hdw@uni-muenster.de (H.-D.W.)

**Keywords:** Polyphosphazene, polymer electrolyte, electrochemical stability, lithium metal battery

## Abstract

A modified polyphosphazene was synthesized using a mixed substitution at phosphorus consisting of 2-(2-methoxyethoxy)ethoxy side groups and anionic trifluoroborate groups. The primary goal was to increase the low lithium ion conductivities of the conventional lithium salt containing poly[2-(2-methoxyethoxy)ethoxy-phosphazene] (MEEP) by the immobilized anionic groups. As in previous studies, the mechanical stability was stabilized by UV induced radiation cross linking. By variation of the molar ratio between different side groups, mechanical and electrochemical properties are controllable. The polymer demonstrated large electrochemical stability windows ranging between 0 and 4.5 V versus the Li/Li^+^ reference. Total and lithium conductivities of 3.6 × 10^−4^ S·cm^−1^ and 1.8 × 10^−5^ S·cm^−1^ at 60 °C were revealed for the modified MEEP. When observed in special visualization cells, dendrite formation onset time and short-circuit time were determined as 21 h and 90 h, respectively, under constant current polarization (16 h and 65 h for MEEP, both with 15 wt % LiBOB), which hints to a more stable Li/polymer interface compared to normal MEEP. The enhanced dendrite suppression ability can be explained by the formation of a more conductive solid electrolyte interphase (SEI) and the existence of F-contained SEI components (such as LiF). With the addition of ethylene carbonate–dimethyl carbonate (EC/DMC) to form MEE-*co*-OBF_3_P gel polymer, both total and lithium conductivity were enhanced remarkably, and the lithium transference numbers reached reasonable values (*σ*_total_ = 1.05 mS·cm^−1^, *σ*_Li_*^+^* = 0.22 mS·cm^−1^, $t_{{\mathrm{Li}}^{+}}$ = 0.18 at 60 °C).

## 1. Introduction

Polymer electrolytes (SPEs) offer improved safety and reliability as compared to electrolytes based on liquid solvents only [1,2,3,4,5,6,7]. An additional advantage might be an enhanced stability towards lithium dendrite formation in contact with lithium metal anodes as was reported by several authors [8,9,10,11,12]. However, the achievable steady-state lithium ion current densities suffer from the very low lithium ion conductivities as well as the low transference numbers, *T*_+_, which typically range between 0.1 and 0.25 [13,14,15].

Therefore, polymer electrolytes are usually transformed to polymer gels by gelification with polar aprotic liquids, which largely enhance the ionic mobility [16,17,18,19]. An additional improvement is expected by the use of polymers with immobilized anions (= polyanions) whose charge is compensated by mobile lithium ions. This strategy should lead to single lithium ion transport and a transference number equal to one. There have been several studies reporting experiences on this concept regarding different polymers [20,21,22,23,24,25]. Allcock et al. was already able to synthesize a polyphosphazene based single ion conductor with arylsulfonimide side groups covalently linked to the polymer backbone. The lithium ion concentration depended on the net percentage of lithiated sulfonamide substituents, but as most often found by these and other authors before, the mobility of ions remained small, reaching 2.45 × 10^−6^ S cm^−1^ at ambient temperatures and 4.99 × 10^−5^ S cm^−1^ at 80 °C [26]. Such values do not yet meet the requirements of practical batteries. Fiedler et al. synthesized the polyphosphazene based polymer electrolytes with cyclic ether side groups, showing an extremely high lithium transference numbers of 0.6, but rather low lithium conductivities of 2.8 × 10^−6^ S cm^−1^ at 60 °C for poly [(1,3-dioxane-5-oxy) (1,3-dioxolane-4-methoxy)-phosphazene] [27].

Recently, we introduced the concept of polymers with immobilized anionic trifluoroborate groups. These are easy to prepare and tend to favor a good dissociation of the charge compensating lithium ions [28]. It is to be expected that the glass transition temperature will increase with the concentration of anionic substituents which, however, cannot be increased to 100% of the phosphorous sites as an increase soon will lead to much less segmental mobility of the polymer and accordingly to a slowing down of the cation mobility. The latter may be partially stopped by adding a liquid solvent to form a polymer gel. However, a compromise is an appropriate ratio of donor substituents with ether oxygens (usual lithium ion solvating groups in ether-based salt-in-polymer electrolytes) and additional more rigid anionic groups (each combined with a free lithium ion). The ratio can be optimized to achieve good ion mobility and lithium ion concentration and hence a favorable partial conductivity of lithium ions.

This work demonstrates the electrochemical properties of a mixed substituted polyphosphazene, which can be used as polymer electrolyte in combination with lithium metal anodes. The parent poly[bis(2-(2-methoxyethoxy)ethoxy)-polyphosphazene] (MEEP) with dissolved salt is already well known as a good polymer electrolyte [29,30,31]. The idea was to replace a moderate part of 5% to 10% of the polyether side chains at the phosphorous by fixed anionic –OBF_3_^−^ groups which should retain the good mechanical and electrochemical properties of MEEP.

## 2. Materials and Methods

### 2.1. Starting Materials

Toluene (99.7%, VWR, Darmstadt, Germany), tetrahydrofurane (99.5%, VWR), sulfuryl chloride (SO_2_Cl_2_, 98%, Aldrich, Munich, Germany), *n*-pentane (98%, Baker, Gross-Gerau, Germany), phosphorous trichloride (PCl_3_, 99%, Merck KGaA, Darmstadt, Germany), and 2-(2-Methoxyethoxy) ethanol (C_5_H_12_O_3_ 99%, Aldrich) were freshly distilled beforehand. Sodium hydride (60% dispersion in mineral oil, Aldrich), sodium hydroxide (98%, Aldrich), *n-*butyl lithium solution (1.6 M in hexane, Aldrich), lithium bis(trimethylsilyl)amide (LiN(Si(CH_3_)_3_)_2_, 97%, Aldrich), boron trifluoride diethyl etherate (BF_3_·O(C_2_H_5_)_2_, Aldrich), and phosphorous pentachloride (PCl_5_, Aldrich) were kept in an argon filled glove box. The dialysis tubes (1.2–1.4 × 10^−4^ g mol^−1^, Reichelt Chemietechnik, Heidelberg, Germany), Celite545^®^ (Merck KGaA), and the molecular sieve 4 Å (VWR) used for polymer purification were cleaned and dried prior to use (140 °C for 48 h). All synthesis steps were carried out using standard vacuum line Schlenk techniques (under inert gas).

### 2.2. Synthesis of Precursor Polymer

**Figure 1:**
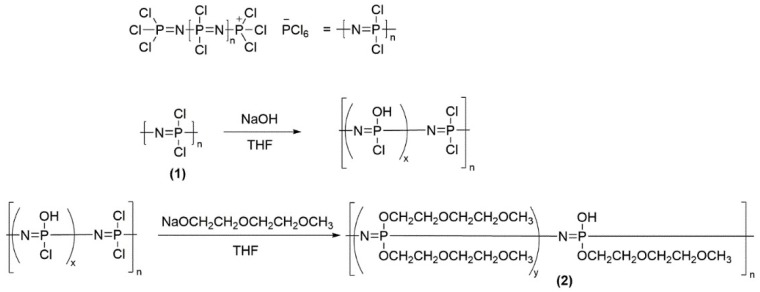
Synthesis of poly[bis 2-(2-methoxyethoxy)ethoxy-*co*-hydroxophosphazene] (**2**).

The precursor polymer, [NPCl_2_]_n_ (**1**), was synthesized based on a synthesis route published by Wang et al. [32,33] and a cationic living polymerization reaction (first published by Allcock et al. [34]) with minor modifications [35]. The intermediate poly[bis 2-(2-methoxyethoxy)ethoxy-*co*-hydroxophosphazene] (**2**) was obtained by nucleophilic substitution of chlorine with a corresponding ratio of sodium alcoholate and sodium hydroxide according to Figure 1. The functionalization of polyphosphazenes with oligoether side chains was already published earlier [14].

**Figure 2:**
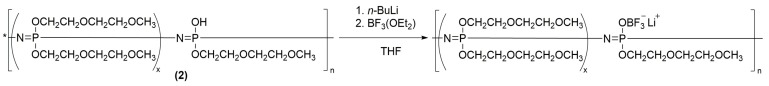
Last step of the synthesis of poly[bis 2(2-methoxyethoxy)ethoxy-*co*-oxytrifluoroborat lithium phosphazene].

The final product, poly[bis 2-(2-methoxyethoxy)ethoxy-*co*-oxytrifluoroborate lithium phosphazene] (**3;4**), was obtained via the synthetic route described in Figure 2. The previously purified and dried poly[bis 2-(2-methoxyethoxy)ethoxy-*co*-hydroxophosphazene] (**2**) was dissolved in dry tetrahydrofurane and cooled to −40 °C in an ice/CaCl_2_ bath. 20 mL of 1.6 M *n*-butyl lithium (in hexane) solution was added dropwise to the polymer solution. The reaction solution was stirred for 1 h at room temperature. After the polymer was dissolved completely, the solution was cooled once again to 40 °C and 5 mL of boron trifluoride diethyl etherate was added dropwise afterwards. The reaction mixture was then stirred for 24 h. The final product poly[bis 2(2-methoxyethoxy)ethoxy-*co*-oxytrifluoroborate lithium phosphazene] (**3;4**) with a specific substitution ratio (5 mol % in polymer **3**, and 10 mol % in polymer **4**) was obtained after being dried under vacuum at 60 °C.

In the following, we use the abbreviations MEE-*co*-OBF_3_Li(5)P and MEE-*co*-OBF_3_Li(10)P for two series of mixed conducting polyphosphazene samples with two different concentrations 5% and 10% of the anionic oxytrifluoroborate groups referred to the total number of substituents. The oxygentrifluoroborate groups were neutralized by an additional number of lithium cations.

### 2.3. Characterization of the Final Polymers

^1^H, ^11^B, ^31^P, and ^13^C NMR spectra were acquired for examination of the product (Bruker AVANCE (III) 400 UltraShield^®^ spectrometer, 400.03 MHz, Rheinstetten, Germany). NMR data were analyzed using Mestrelab Research S.L. MestReNova v10.0.0-14381.

The polymer was analyzed via gel permeation chromatograph (GPC) (Polymer Standards Service GmbH, Mainz, Germany) to obtain the molecular weight and distribution. The detector was calibrated in advance based on polystyrene standards (Ready-Cal standard, Polymer Standards Service GmbH, Mainz, Germany). The obtained GPC data were processed using PSS WINGPC (built 5403) software (Polymer Standards Service GmbH, SR1).

Investigation of thermal stability was done using DSC (NETZSCH DSC 204, Selb, Germany), data analysis was performed with NETZSCH software (v6.1.0). 5–10 mg of the as-synthesized polymer was sealed into an aluminum can and measured from −150 to 250 °C with a heating rate of 10 °C/min. The heating cycle was repeated for another two times and the last cycle was used for interpretations.

The total ionic conductivity was measured via AC impedance spectroscopy (frequency response analyzer E 4980 A, Agilent Technologies, Waldbronn, Germany) in a frequency range from 20 Hz to 2 MHz. A peak-to-peak voltage amplitude of 40 mV was used. The polymer membranes were sandwiched between two stainless steel electrodes (1 cm^2^). The measurement data were recorded in a temperature range from −20 to 90 °C, controlled by a Julabo FP45 thermostat.

Electrochemical stability windows were measured by cyclic voltammetry in three-electrode Swagelok^®^ cells at 60 °C with a scan rate of 1 mV·s^−1^ (Autolab PGSTAT302N, Metrohm, Filderstadt, Germany). The as-prepared salt-in-polymer membranes (thickness 200 μm) were cut into circular discs (area = 0.785 cm^2^). Lithium foil was employed as both the reference electrode (area = 0.393 cm^2^) and the counter electrode (area = 0.785 cm^2^). The cathodic electrode potential ranges (2.5 down to −0.5 V, against Li/Li^+^) were measured with a Cu working electrode (area = 0.785 cm^2^) while the anodic electrode potential ranges (2.5 to 7 V) were monitored with a Pt working electrode (0.785 cm^2^).

The lithium ion transference numbers of the as-prepared polymer electrolytes were determined via a method as described by Hiller et al. using two-electrode Swagelok^®^ cells [36].

Impedance spectra for polymer electrolytes in symmetric cells with two lithium metal electrodes were measured using a combination of the Solartron SI 1287 potentiostat with a Solartron SI 1260 impedance analyzer in the frequency range of 1 mHz to 1 MHz with a peak-to-peak amplitude of 10 mV.

### 2.4. Preparation of Solid Polymer Electrolyte Membranes

The as-synthesized polymers (**3,4**) were dried under vacuum at 60 °C for 1 week before use. Subsequently, around 1 g of the dried polymer was dissolved in freshly distilled THF. 10 wt % of benzophenone and 15 wt % of lithium salt were added to the solution afterwards. The solution was stirred until benzophenone and the lithium salt were dissolved completely. Then, the vessel was transferred to an oven and dried under vacuum at 60 °C for 24 h until THF was removed completely. To prepare thin membranes of the salt-in-polymer electrolyte, a suitable amount of the dried mixture was sandwiched between two Mylar^®^-foils and pressed to a required thickness (200–300 µm). Finally, salt-in-polymer membranes were obtained after cross-linking by applying UV-irradiation for 12 min. A series of polymer electrolyte membranes were obtained in the same way with different lithium salts (5/10 mol % -OBF_3_Li substituted polymers with 15 wt % LiBOB, LiTFSI, and LiFSI).

### 2.5. Preparation of Gel Polymer Electrolyte Membranes

The as-synthesized polymers were mixed thoroughly with a calculated amount of lithium salt, ethylene carbonate–dimethyl carbonate (EC/DMC, 1:1), and benzophenone (Table S1). Subsequently, the mixture was sandwiched between two Mylar^®^-foils and pressed to a required thickness. The intended gel polymer electrolyte membranes (polymer **5**) were obtained after cross-linking by applying UV-irradiation for 12 min, with intermediate cooling after each minute (by dry ice). Based on our previous experience, MEEP/LiBOB gel polymer demonstrated better cyclability and possessed a more stable Li/MEEP interface as compared to gels with other lithium salts. It is evident that the bis-oxalatoborate anions support the formation of a stable and dense SEI [31,37]. Therefore, only LiBOB was used for the preparation of gel polymer electrolytes in this work.

### 2.6. Cell Set Up

A transparent cell was combined with an optical microscope to monitor the formation and evolution of the lithium dendrites on the lithium/polymer electrolyte interface. The cell configuration is shown in Figure S1, where two narrow lithium metal strips attached to the aluminum conductor were placed on opposite areas of the composite polymer electrolyte membrane, the latter with a thickness of around ~200 µm [37]. All these assembled cell components were then put between two glass plates with a thickness of 1 mm, and further covered by PEEK to keep any gas atmosphere away from the lithium. All measurements were carried out in a dry room.

## 3. Results and Discussion

### 3.1. Polymer Characterization

For the as-prepared polymer, an average molecular weight of 1.5 × 10^5^ Da and a polydispersity index (PDI) of 1.32 were determined by the GPC measurement. Two peaks appeared in the ^31^P-NMR spectra (Figure S2) of polymer (**4**), corresponding to di-(2-2-methoxyethoxy)ethoxy) substituted phosphorous (−7.35 ppm) and mono-(2-(2-methoxyethoxy)ethoxy)/mono-(lithium trifluoro borate)oxy substituted phosphorous (−13.53 ppm), respectively (corresponding ^1^H NMR spectra shown in Figure S3). The OBF_3_-Li substitution ratio is quantified to be 12%, by simply comparing the areas of integrated peaks at −7.35 and −13.53 ppm. ^19^F-NMR spectra of both polymer (**4**) and LiTFSI with a molar ratio of 1:1 are shown in Figure S4, the areas of these two observed peaks in the spectra were also used to confirm the complete reaction, indicating that the OBF_3_-Li substitution ratio is around 8%. By ^19^F-NMR spectroscopy, the –OBF_3_ functional group was identified with a peak at −155.8 ppm. These values are typical for F-atoms in R–O–BF_3_ species [38]. For ^11^B-NMR spectra (Figure S5), one peak was shown at −1.00 ppm. 

### 3.2. Thermal Properties

**Figure 3:**
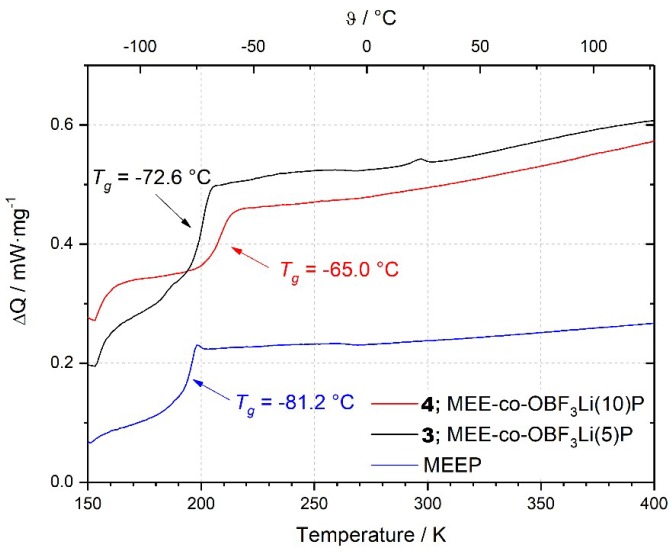
DSC Measurements of MEEP, MEE-*co*-OBF_3_Li(5)P, and MEE-*co*-OBF_3_Li(10)P.

Figure 3 shows the DSC heating curves of three different cross-linked polymers. It indicates that the introduction of anionic species leads to stronger ionic intermolecular interactions and decreases the segmentally movement of oligoether sidechains, since both polymer **3** and **4** exhibit higher *T_g_* values as compared to MEEP, especially for polymer **4** with a higher substitution ratio. Nevertheless, all polymers shown here are thermally stable up to at least 150 °C.

### 3.3. Ionic Conductivity and Electrochemical Stability

**Figure 4:**
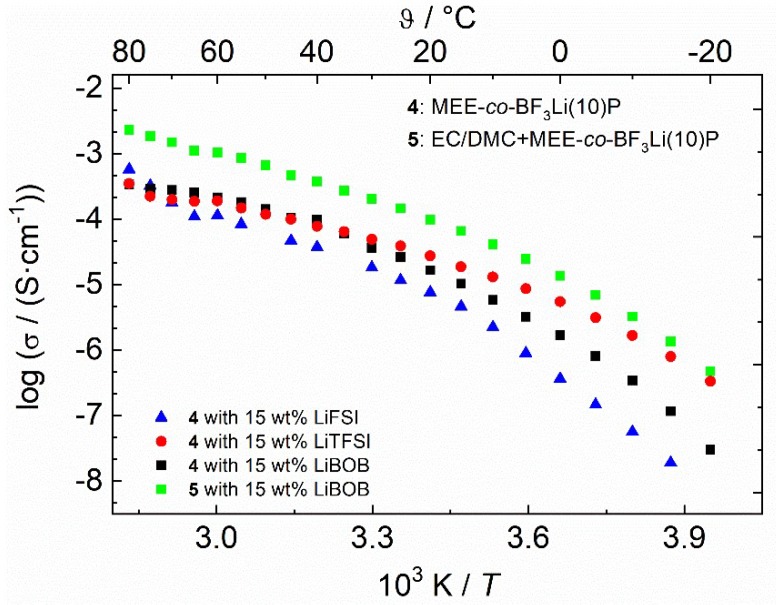
Arrhenius-plot of the cross-linked polymer **4** and **5** membranes with different lithium salts (15 wt %).

Table 1 summarizes the ionic conductivities of selected polymers at certain temperatures. The ionic conductivities of the pure polymers **3** and **4** without additionally dissolved lithium salts are 3.2 × 10^−6^ and 2.5 × 10^−6^ S·cm^−1^, respectively, at 25 °C, which are well comparable to other modified MEEP based polymers reported in the literature (2.5 × 10^−6^ S·cm^−1^ as reported by Allcock; 5 × 10^−9^ S·cm^−1^ as reported by Fiedler, both at 25 °C) [26,27,39]. LiTFSI, LiFSI, and LiBOB were added to the as-prepared polymers to produce salt-in-polymer electrolyte membranes. As shown in Figure 4, polymer **4** with LiTFSI exhibits the highest ionic conductivity in the low temperature range (4.9 × 10^−5^ S·cm^−1^ at 30 °C). While at high temperature ranges, polymer with LiFSI shows a better $\sigma_{total}$ of 5.0 × 10^−4^ S·cm^−1^ at 80 °C. After adding EC/DMC to the dry polymer, the total conductivity is enhanced by almost one order of magnitude. The total conductivity of polymer **5** with LiBOB reaches 2.5 × 10^−3^ S·cm^−1^ at 80 °C.

The contribution of lithium ions to the total conductivity, i.e. the partial lithium ion conductivity corresponds to the product of the total conductivity and the lithium transference number:

(1) $$\sigma_{{\mathrm{Li}}^{+}}=T_{+}\cdot \sigma_{\mathrm{total}}$$

**Figure 5:**
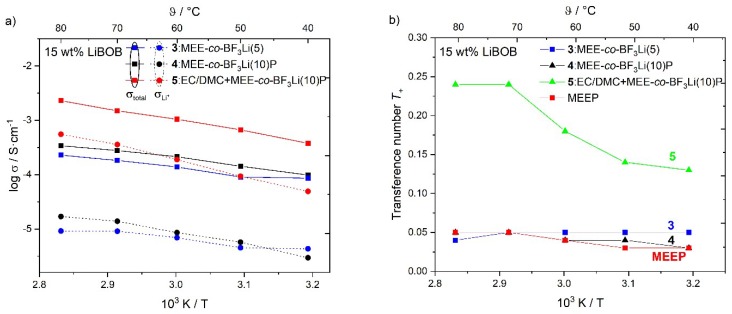
(**a**) Arrhenius diagram for cross-linked polymer **3**, **4**, and **5** with 15 wt % LiBOB showing the total and partial lithium conductivity (σ_total_ and *σ*_Li_*^+^*). (**b**) Corresponding lithium transference numbers of **3**, **4**, **5**, and MEEP.

Figure 5a shows the Arrhenius-type plots of the temperature dependent total conductivity, *σ*_total_, and the partial lithium conductivity, *σ*_Li_*^+^*, of polymers **3**,**4**, and **5** each containing 15 wt % dissolved LiBOB, while Figure 5b plots the corresponding lithium ion transference numbers, *T_+_*. With a higher substitution ratio, polymer **4** shows better total and lithium conductivity compared to polymer **3** at almost all measured temperatures (except *σ*_Li_*^+^* at 40 °C). However, for both polymer electrolytes **3** and **4**, the obtained lithium transference numbers show similar values lower than 0.1, which are lower than PEO (typically around 0.15) [15]. Desirably, the introduction of the OBF_3_-Li group would contribute to more mobile lithium ions, thus leading to a higher concentration of the free charge carrier and increasing lithium conductivity. However, the determined lithium transference number is only slightly better than MEEP salt-in-polymers at lower temperatures [14].

**Figure 6:**
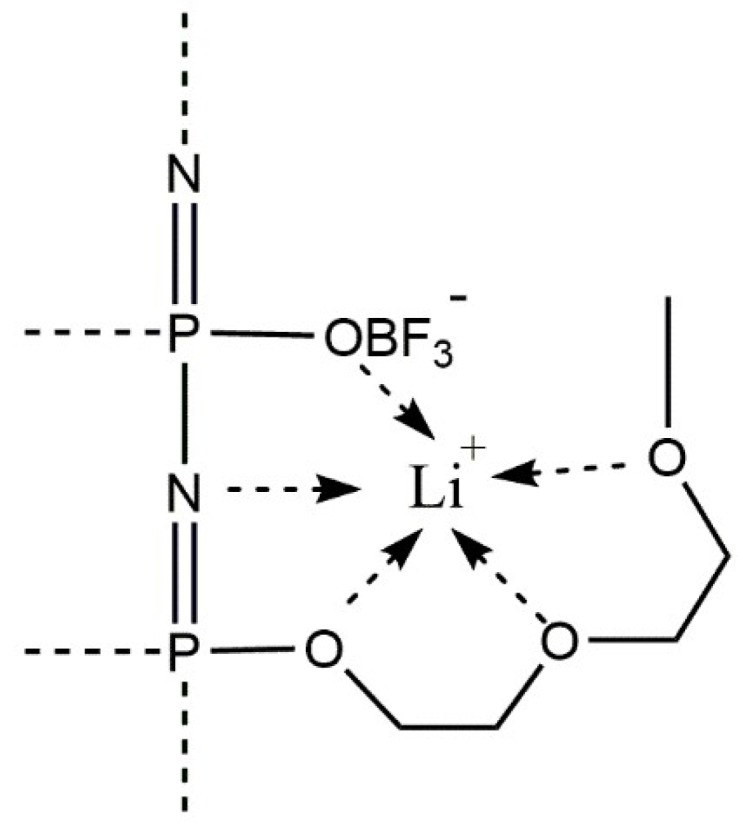
Illustration of coordination sites of the lithium cations by ethylene oxide sidechains, the anionic substituents and the basic nitrogen atoms in the backbone.

The low lithium transference number is due to an enhanced coordination of the lithium cations by the oligoethylene oxide segments of the side chain and by the lone electron pair of the basic nitrogen atoms of the backbone [14,40]. This so-called “pocket effect” inhibits the mobility of lithium cations (see Figure 6) and could only be circumvented by a much larger concentration of (OBF_3_)^−^ substituents along the polymer backbone. Lee et al. also discussed the strong interaction between the cations and the polymer as limiting the cation contribution to the overall conductivity [41]. Furthermore, as is demonstrated in Table 1, only a small molar increase of the total lithium ion concentration is achieved by the additional lithium counterions of the anionic groups. Therefore, further increasing the amount of substituted group or lithium salt concentration becomes necessary. Nevertheless, the salt in MEE-*co*-OBF_3_LiP membranes show good ionic conductivity at elevated temperatures ~90 °C with no indication of degradation, which indicates that such polymer electrolyte membranes have better thermal stability as compared to conventional LiPF_6_ based organic electrolytes, especially when operating at temperatures higher than 60 °C [42].

The addition of EC/DMC results in partial lithium ions being coordinated and transported by the carbonates in the liquid electrolyte phase [31], thus increasing the lithium transference number. The *T*_+_ value for EC/DMC+MEE-*co*-OBF_3_Li(10)P/LiBOB reaches 0.24 (70 °C, gel polymer), which is better than the dry salt in PEO electrolytes [15]. Hence, along with the much better total conductivity, the lithium ion conductivity of MEE-*co*-OBF_3_Li(10)P/LiBOB gel polymer is enhanced to 3.6 × 10^−4^ S·cm^−1^ at 70 °C.

To analyze the practicability of the as-prepared polymers in combination with the lithium metal anode, lithium plating/stripping experiments for polymer **4** and **5** with different salts were performed. As is demonstrated in Figure S6, both the dry and gel polymers exhibit a relatively stable overpotential versus time profile and show no sign of severe electrolyte degradation.

**Figure 7:**
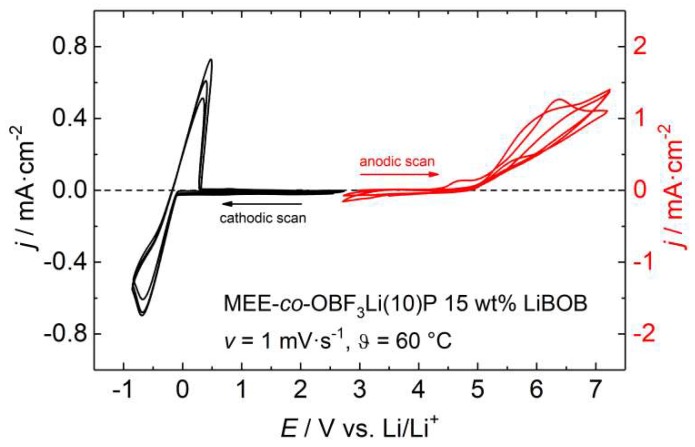
Cyclic voltammogram of MEE-*co*-OBF_3_Li(10)P with 15 wt % LiBOB at 60 °C. Working electrode: Cu (for the cathodic scan on the left) and Pt (for the anodic scan on the right).

Figure 7 shows the cyclic voltammogram of several anodic and cathodic cycles of polymer **4** with 15 wt % LiBOB salt at 60 °C. An anodic oxidation is evident by an increase of current density beginning at 4.5 V for the electrolyte MEE-*co*-OBF_3_Li(10)P/LiBOB, which agrees with the usually reported anodic stability referred to Li^+^/Li for PEO based electrolytes [43,44] and is caused by irreversible oxidation within the polyether chains [14,45]. The reversible lithium plating/stripping process becomes visible in the corresponding cathodic scan (around 0 V). Maximum current densities of −0.80 mA·cm^−2^ during lithium deposition and 0.64 mA·cm^−2^ during dissolution yield a coulombic efficiency of 80%. The loss of lithium indicates the formation of a SEI.

### 3.4. Analysis of Conditions for Li Dendrite Formation at the Li/MEE-co-OBF_3_LiP (+LiBOB) Interface

**Figure 8:**
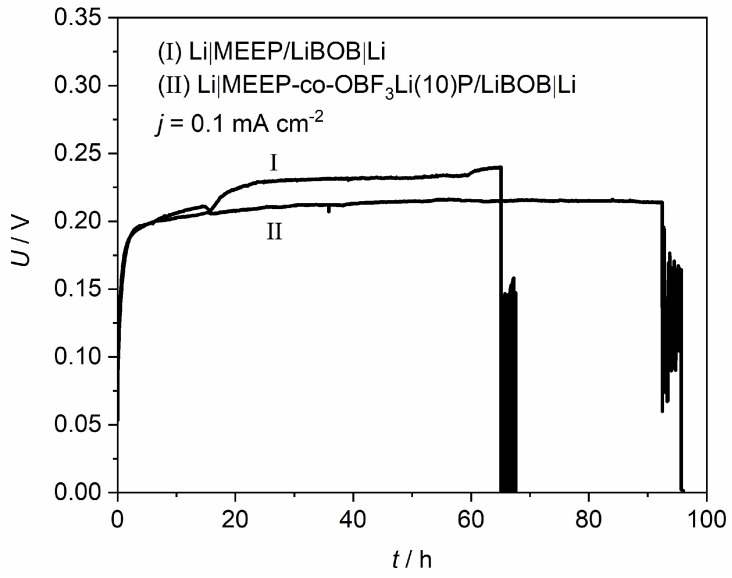
Constant current polarization curve of (I) Li|MEEP/LiBOB|Li and (II) Li|MEE-*co*-OBF_3_P/LiBOB|Li at 0.1 mA·cm^−2^, 60 °C.

**Figure 9:**
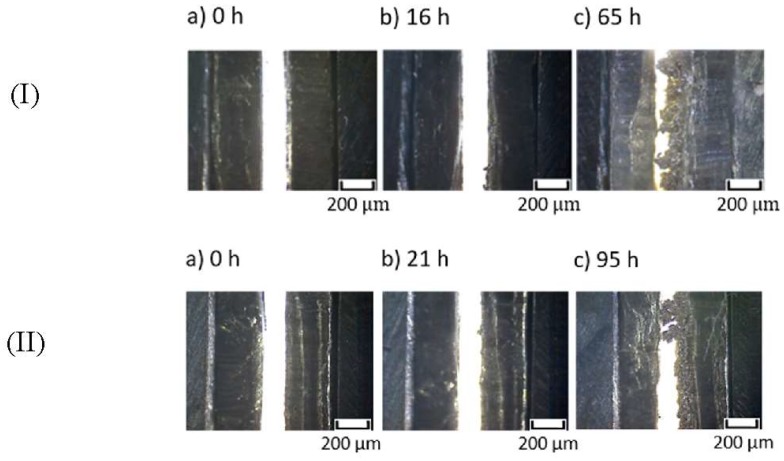
Dendrite growth in (**I**) Li|MEEP/LiBOB|Li and (**II**) Li|MEE-*co*-OBF_3_Li/LiBOB|Li visualization cell at 60 °C and 0.1 mA·cm^-2^; (**a**) before polarization; (**b**) dendrite structures observed; (**c**) dendrite reached positive electrode and short-circuit the cell.

To further investigate the effect of the introduced –OBF_3_Li group on the interfacial stability, comparative studies of modified and unmodified MEEP were carried out in lithium symmetric cells with regard to reversibility and stability of lithium deposition under dc current flow. Figure 8 demonstrates the polarization curves for MEEP and MEE-*co*-OBF_3_Li(10)P (both with 15 wt % LiBOB) in the visualization cell at a constant current density of 0.1 mA/cm^2^. A slightly lower overpotential is observed for MEE-*co*-OBF_3_Li(10)P, which suggests a lower cell impedance. A clear short-circuit is observed after a time of 65 h for the unmodified MEEP (I), corresponding to an abrupt voltage drop due to short circuit. This interpretation is confirmed by our microscope observation in Figure 9Ic, which marks the appearance of non-removable short-circuits by lithium dendrites. In addition, dendrite formation onset time (t_0_) is determined as 16 h from Figure 9Ib. Similarly, the dendrite formation onset time and short-circuit time are determined as 21 and 90 h, respectively, for MEE-*co*-OBF_3_P/LiBOB; both appear to be longer as compared to MEEP/LiBOB dry polymer.

**Figure 10:**
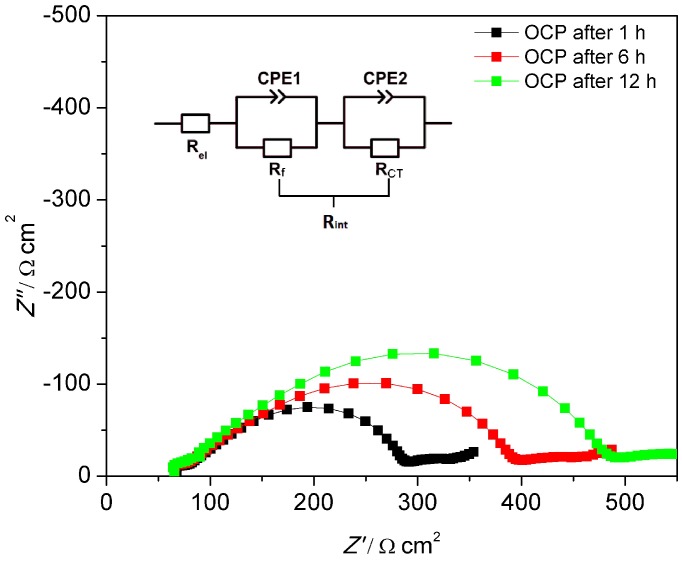
Impedance spectra of Li|MEEP/LiBOB|Li at 60 °C after 1, 6, and 12 h under OCP. The table lists the corresponding R_el_, R_f_, and R_CT_ at different stages. R_el_ is the electrolyte bulk resistance and R_int_ is the interface resistance, which consists of R_f_ and R_CT_.

**Figure 11:**
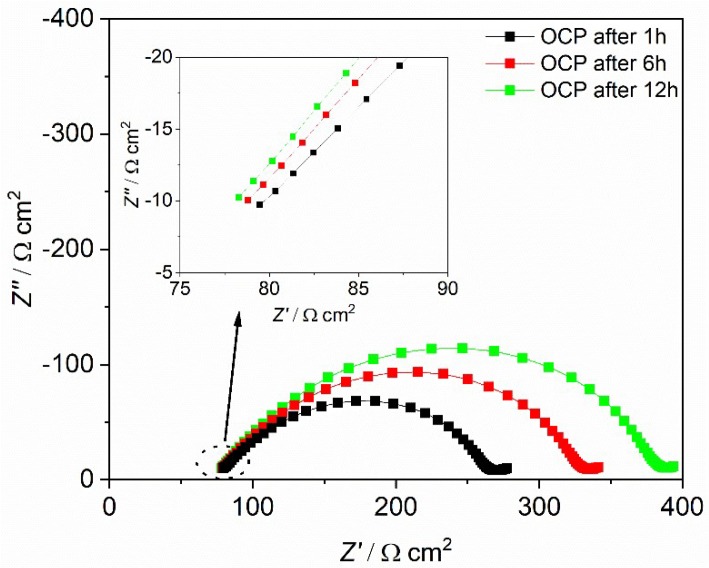
Impedance spectra of Li|MEE-*co*-OBF_3_P/LiBOB|Li at 60 °C after 1, 6, and 12 h under OCP conditions. The table lists the corresponding R_el_, R_f_, and R_CT_ at different stage. R_el_ is the electrolyte bulk resistance and R_int_ is the interface resistance, which consists of R_f_ and R_CT_.

To explain this interesting difference between unmodified MEEP and BF_3_-modified MEEP, impedance spectra are shown in Figure 10 and Figure 11 for MEEP and MEE-*co*-OBF_3_P, respectively, each with 15 wt % LiBOB measured in the symmetric cells under open circuit potential condition.

The impedance spectra were analyzed employing ZView with the equivalent circuit displayed in Figure 10. In accordance to other polymer electrolytes in the literature [37,46,47], the high frequency intercept with the Z’ axis in the left can be assigned to the polymer electrolyte bulk resistance (R_el_), yielding its bulk conductivity. The diameter of the large distorted semicircle can be ascribed to the interfacial resistance (R_int_), which can be interpreted as two partly overlapping semicircles. The latter may be represented by a series of two different impedance contributions each consisting of a resistance element in parallel to a constant phase element. As is shown in Figure 10, these two overlapping semicircles with similar capacities can be attributed to the passivation film resistance from the solid electrolyte interface (R_f_) and the charge transfer resistance resulting from the transfer and deposition of lithium ions at the lithium metal interface (R_CT_). The inclined tail of the spectrum (on the low frequency end on the right) contains the impedance due to ambipolar salt diffusion during formation of concentration gradients at very small frequencies, i.e. ω→0.

For both polymer electrolytes, the bulk resistances stay almost constant during the whole resting period. R_el_ for MEEP/LiBOB is slightly lower compared to MEE-*co*-OBF_3_LiP/LiBOB, due to the slightly better total ionic conductivity of MEEP/LiBOB. On the other hand, the interface resistance dominates the cell impedance for both polymers and keeps increasing with the storage time in the measured cell, which reflects a continuous growth of the SEI at the interface, since the R_CT_ varies only slightly for both polymers. Comparison of these two figures also hints to a more conductive SEI at the Li/MEE-*co*-OBF_3_LiP interface. After 12 h of storage under the OCP condition (which is also the rest-time for cells before polarization), R_f_ for MEE-*co*-OBF_3_LiP/LiBOB reaches 203 Ω cm^2^, which is almost 40% lower than MEEP/LiBOB (316 Ω cm^2^). An enhanced interfacial transport of Li^+^ (lower R_int_) will postpone the time point when the cation concentration drops to zero at the anode and increase the onset time of dendrite formation according to Sand’s model [37,46,48,49]. In addition, previous works in the literature have already shown that a faster lithium ion migration in the SEI (lower R_f_) would stabilize lithium electrodeposition [47,50,51]. This then gives an explanation for the better dendrite suppression effect of MEE-*co*-OBF_3_P/LiBOB as compared to MEEP/LiBOB. Moreover, the introduced –OBF_3_ group may act as a fluoride ion source similar to the PF_6_^-^ anion in classic liquid lithium ion battery electrolytes as suggested by Grünebaum et al. [28]. This could result in the formation of a SEI component containing solid LiF, which was known to be able to suppress dendrite growth due to the higher surface energy and lower surface diffusion barrier for adatoms (faster diffusion along the surface) [52].

## 4. Conclusions

In this work, we present a new type of polyphosphazene based polymer electrolyte. The introduction of boron trifluoride as the anionic species to the polyphosphazene backbone was successful. The solid polymer electrolyte membranes exhibit maximum *σ*_total_ and *σ*_Li_*^+^* of 3.6 × 10^−4^ S·cm^−1^ and 1.8 × 10^−5^ S·cm^−1^, respectively, at 60 °C, which are better than the recently reported polyphosphazene based polymer electrolytes in the literature [26,27], and comparable to other salt in polymer electrolytes, like PEO:LiTFSI [36,39]. With the addition of EC/DMC, MEE-*co*-OBF_3_P gel polymer shows enhanced total and lithium conductivity, accompanied with a much higher lithium transference number (*σ*_Li_*^+^* = 0.36 mS·cm^−1^, *T*_+_ = 0.24 at 70 °C), which makes MEE-*co*-OBF_3_P gel polymer a promising electrolyte for further study.

Though a similar lithium transference number was observed as compared to MEEP/LiBOB dry polymer, MEE-*co*-OBF_3_P/LiBOB exhibits enhanced dendrite suppression ability due to the formation of more conductive SEI and the existence of F-contained SEI components (such as LiF).

Few approaches are available to further enhance the lithium conductivity of the modified MEEP, such as introducing a higher amount of substituted anion group (–OBF_3_Li) or lithium salts into the polymer structure to enable the lithium ion hopping mechanism, and introducing Mg^2+^ to preferentially coordinate with ethylene oxide sidechains, which increases the amount of mobile Li^+^ ions.

## Figures and Tables

**Table:** 

MEEP/LiBOB (Ω cm^2^)			
	R_el_	R_f_	R_ct_
OCP, 1 h	54	163	104
OCP, 6 h	53	238	109
OCP, 12 h	52	316	120

**Table:** 

MEE-*co*-OBF_3_LiP/LiBOB (Ω cm^2^)			
	R_el_	R_f_	R_ct_
OCP, 1 h	72	101	97
OCP, 6 h	72	155	109
OCP, 12 h	71	203	114

**Table 1:** Summarize of main properties of as-prepared polymers in this paper.

	*σ*_tot_ / [mS·cm^−1^] 25 °C	*σ*_tot_ / [mS·cm^−1^] 60 °C	*σ*_Li_^+^ / 10^−3^ [mS·cm^−1^] 60 °C	T_+_ 60°C	T_g_	*n*(Li_Salt_):*n*(O_EO_)	*n*(Li_tot_):*n*(O_EO_)	[P=N]:O
**(3) MEE-*co*-OBF_3_(5)LiP**					−72.6			
+LiBOB	0.038	0.138	5.52	0.04	−49.7	1:26	1:21	1:5.7
+LiFSI	0.017	0.113	5.65	0.05	−49.9	1:25	1:21	1:5.7
+LiTFSI	0.037	0.171	5.13	0.03	−57.4	1:39	1:29	1:5.7
**(4) MEE-*co*-OBF_3_(10)LiP**					−65.0			
+ LiBOB	0.052	0.356	17.80	0.05	−53.7	1:26	1:18	1:5.7
**(5) EC/DMC+ MEE-*co*-OBF_3_(10)LiP**								
+ LiBOB	0.15	1.05	220	0.18	-	1:26	1:18	1:5.7
**MEEP**					−81.2			
+LiBOB	0.023	0.553	22.10	0.04	−45.9	1:27	1:27	1:6

## Supplementary Materials

The following are available online at http://www.mdpi.com/2073-4360/10/12/1350/s1, Figure S1, Configuration of visualization cell, reprinted from He et al. [37], Figure S2, ^31^P NMR (376 MHz, d^8^-THF, 300 K) of MEE-*co*-OBF_3_LiP, (polymer **4**), Figure S3, ^1^H NMR (376 MHz, d^8^-THF, 300 K) of MEE-*co*-OBF_3_Li, Figure S4, ^19^F NMR (376 MHz, d^8^-THF, 300 K) of MEE-*co*-OBF_3_LiP (polymer **4** with same molar LiTFSI as comparison), Figure S5, ^11^B NMR (400 MHz, CDCl_3_, 300 K) of MEE-*co*-OBF_3_LiP, Figure S6, Plating/stripping experiments of (a) Li|MEE-*co*-OBF_3_LiP/LiBOB|Li (b) Li|MEE-*co*-OBF_3_LiP/ LiFSI|Li (c) Li|MEE-*co*-OBF_3_LiP/ LiTFSI|Li at 0.01 mA cm^−2^ and (d) Li|MEE-*co*-OBF_3_LiP/LiBOB|Li , (e) gel polymer Li|EC/DMC+MEE-*co*-OBF_3_LiP/LiBOB|Li, at 0.1 mA cm^−2^. 15 wt % corresponding salts were used in all polymer electrolytes, Table S1, Composition of the prepared gel polymer electrolytes based on MEE-*co*-OBF_3_LiP.
