# Supplementary material for: Boron Trifluoride Anionic Side Groups in Polyphosphazene Based Polymer Electrolyte with Enhanced Interfacial Stability in Lithium Batteries
Source: Polymers (Basel). 2018 Dec 5;10(12):1350. doi: 10.3390/polym10121350 (PMC6401971; doi:10.3390/polym10121350)
Supplement: Supplementary file 1 [file polymers-10-01350-s001.pdf]

Boron Trifluoride Anionic Side Groups in Polyphosphazene Based Polymer Electrolyte with Enhanced Interfacial Stability in Lithium Batteries

Sebastian Schmohl †, Xuan He †,* and Hans-Dieter Wiemhöfer

Institute of Inorganic and Analytical Chemistry, University of Münster, Corrensstraße 28/30, 48149 Münster, Germany
** This work was financially supported by the NRW Graduate School of Chemistry
† These authors contributed equally to this work
* Correspondence: x_he0002@uni-muenster.de

Supplementry information:

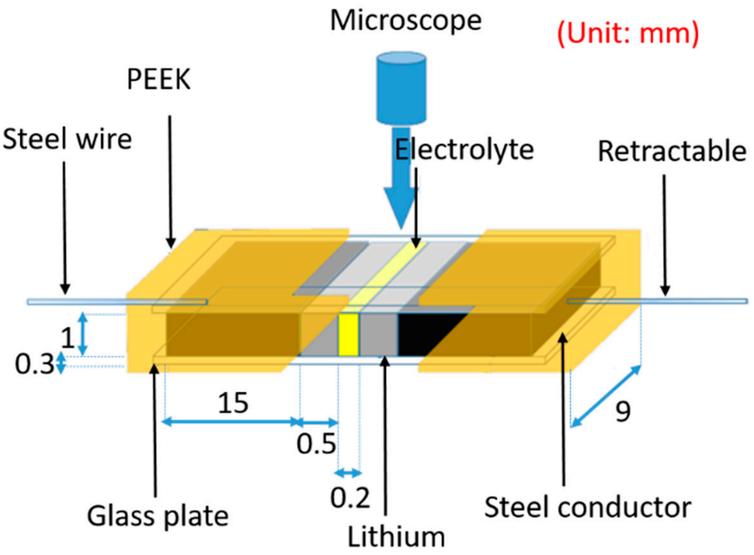

Figure S1: Configuration of visualization cell, reprinted from He et al[1].

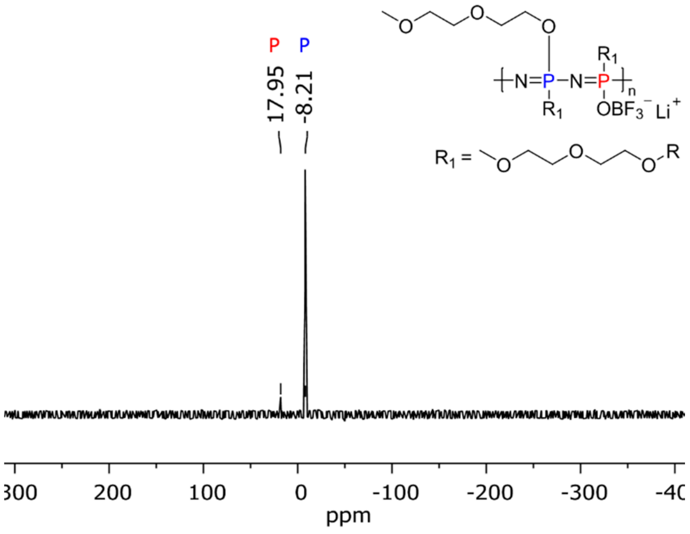

Figure S2: ^{31}P NMR (376 MHz, d^8-THF, 300 K) of MEE-co-OBF_3LiP, (polymer 4)

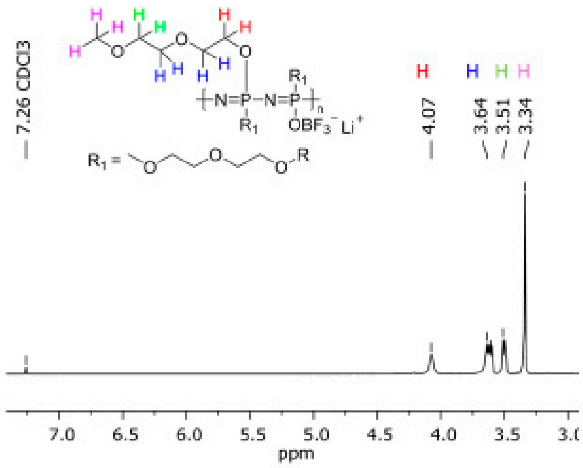

Figure S3:^{1}H NMR (376 MHz, d8-THF, 300 K) of MEE-co-OBF₃LiP

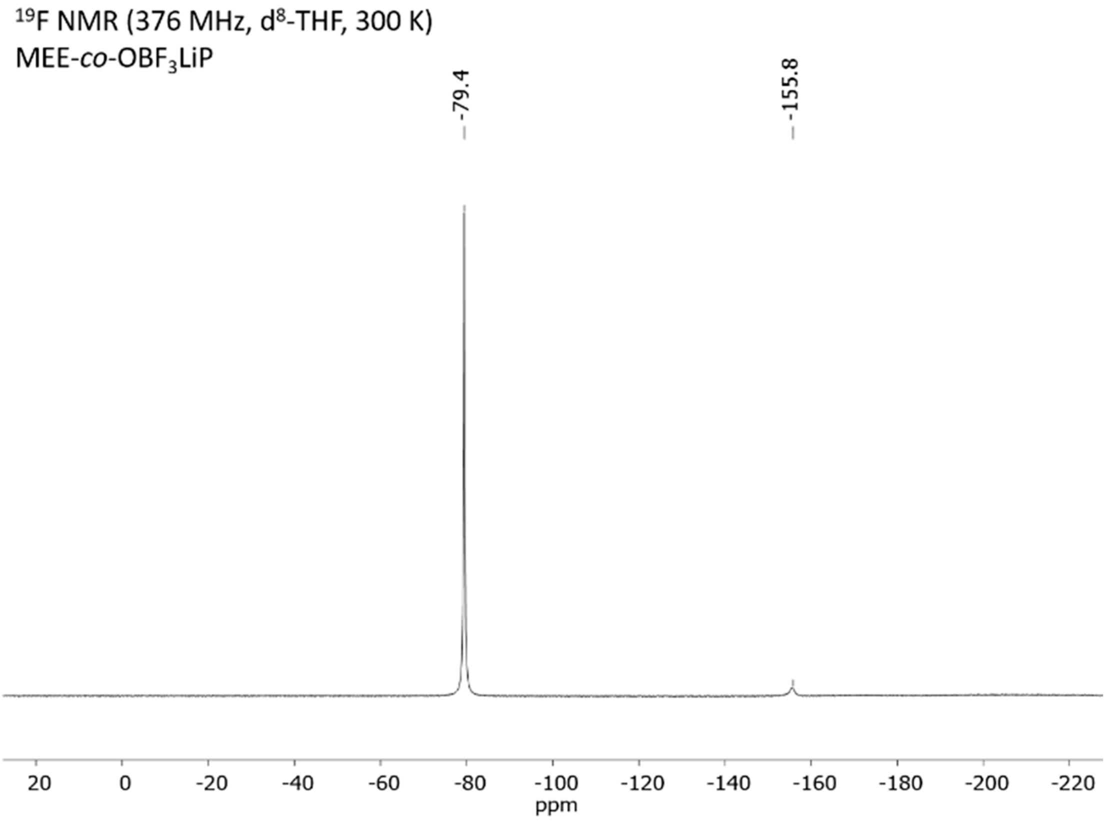

Figure S4:^{19}F NMR (376 MHz, d[8]-THF, 300 K) of MEE-co-OBF_3LiP (polymer 4 with same molar LiTFSI as comparison).

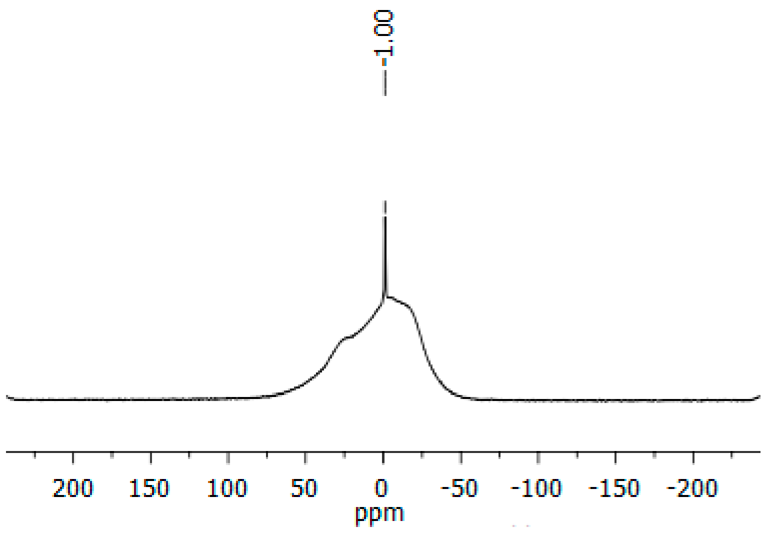

Figure S5: ^{11}B NMR (400 MHz, $CDCl_3$, 300 K) of MEE-co-OBF_3LiP.

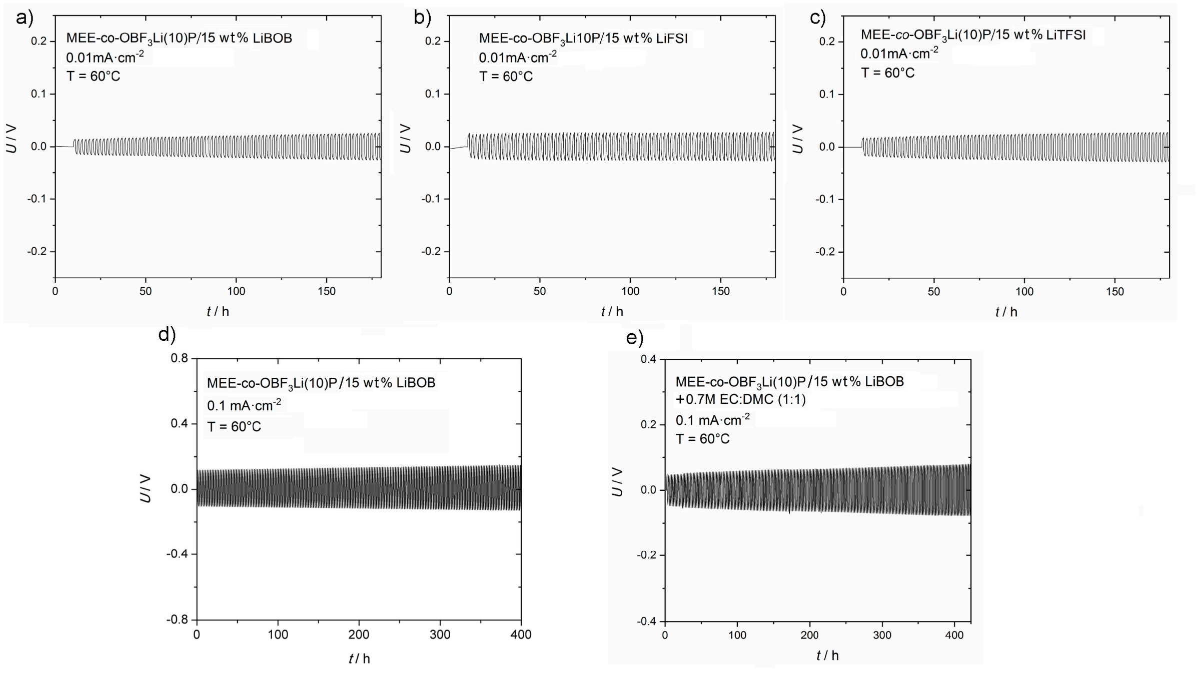

Figure S6: Plating/stripping experiments of (a) Li|MEE-co-OBF_3LiP/LiBOB|Li (b) Li|MEE-co-OBF_3LiP/ LiFSI|Li (c) Li|MEE-co-OBF_3LiP/ LiTFSI|Li at 0.01 mA cm^{-2} and (d) Li|MEE-co-OBF_3LiP/LiBOB|Li , (e) gel polymer Li|EC/DMC+MEE-co-OBF_3LiP/LiBOB|Li,at 0.1 mA cm^{-2}. 15 wt% corresponding salts were used in all polymer electrolytes.

Table S1: Composition of the prepared gel polymer electrolytes based on MEE- co-OBF_3LiP

	**MEE-co-OBF_3LiP**	**LiBOB**	**EC:DMC (1:1)**	**σ_{total} (30°C) / mS·cm^{-1}**
wt%	59.5	9.1	31.4	0.21

1. He, X.; Schmohl, S.; Wiemhöfer, H.-D. Direct observation and suppression effect of lithium dendrite growth for polyphosphazene based polymer electrolytes in lithium metal cells. *ChemElectroChem* **2018**, *Accepted*, doi:10.1002/celc.201801383R2.
